# Metastatic basal cell carcinoma of buccal mucosa: a report of a rare case

**DOI:** 10.1186/s12957-022-02592-4

**Published:** 2022-04-21

**Authors:** Taku Kimura, Ken-ichiro Sakata, Jun Sato, Chisato Ouchi, Noritaka Ohga, Aya Yanagawa-Matsuda, Kyoko Hida, Yoshimasa Kitagawa

**Affiliations:** 1 Department of Oral Diagnosis and Medicine, Hokkaido University Graduate School of Dental Medicine, Sapporo, Japan; 2 Vascular Biology and Molecular Pathology, Hokkaido University Graduate School of Dental Medicine, Sapporo, Japan

**Keywords:** Basal cell carcinoma, Oral metastasis, Intraoral basal cell carcinoma

## Abstract

**Background:**

Basal cell carcinoma (BCC) is the most common cancer worldwide. Most of BCCs can be detected in the early stages and are generally well controlled with local resection. Despite the high incidence of BCC, metastasis is rarely observed. Metastatic BCCs generally have an aggressive phenotype and are refractory to conventional treatment.

**Case presentation:**

We describe a rare case of BCC in which a series of local relapses culminated in metastasis into the oral cavity 10 years after the first diagnosis of cutaneous BCC. We performed surgical resection and postoperative radiotherapy in this patient; 11 months after the final course of radiotherapy, the BCC remains stable, and the patient continues to be monitored regularly.

**Conclusions:**

Because metastatic BCC is refractory to current treatment and difficult to control, his treatment history and the pathohistological features of BCC had to be considered in posttreatment planning.

## Background

Basal cell carcinoma (BCC) is the most common cutaneous cancer. It accounts for approximately 80% of all cutaneous cancers [1], and the incidence is increasing all over the world as a result of ultraviolet irradiation, as well as the ageing of the population [2]. Despite the high incidence, metastasis is rare (approximately 0.0028–0.55% of cases) [3]. Metastasis that does occur is common in the regional lymph nodes, lungs and bone [3]. BCC in the oral cavity, known as intraoral BCC, is extremely rare; the pathohistological findings are similar to those of basaloid squamous cell carcinoma (basaloid SCC), peripheral ameloblastoma and unique odontogenic neoplasms. Intraoral BCCs are most commonly reported in the gingiva, and they manifest with surface ulceration or erythroplakia. BCC in the oral cavity is difficult to diagnose because of its pathohistological similarity to the diseases mentioned earlier. Recently, some immunohistochemistry markers, such as Ber-EP4 and Bcl-2 protein, which are specific markers of BCC cells, have helped establish the diagnosis of BCC. We describe a rare case of metastatic cutaneous BCC in the oral cavity.

## Case presentation

**Fig. 1:**
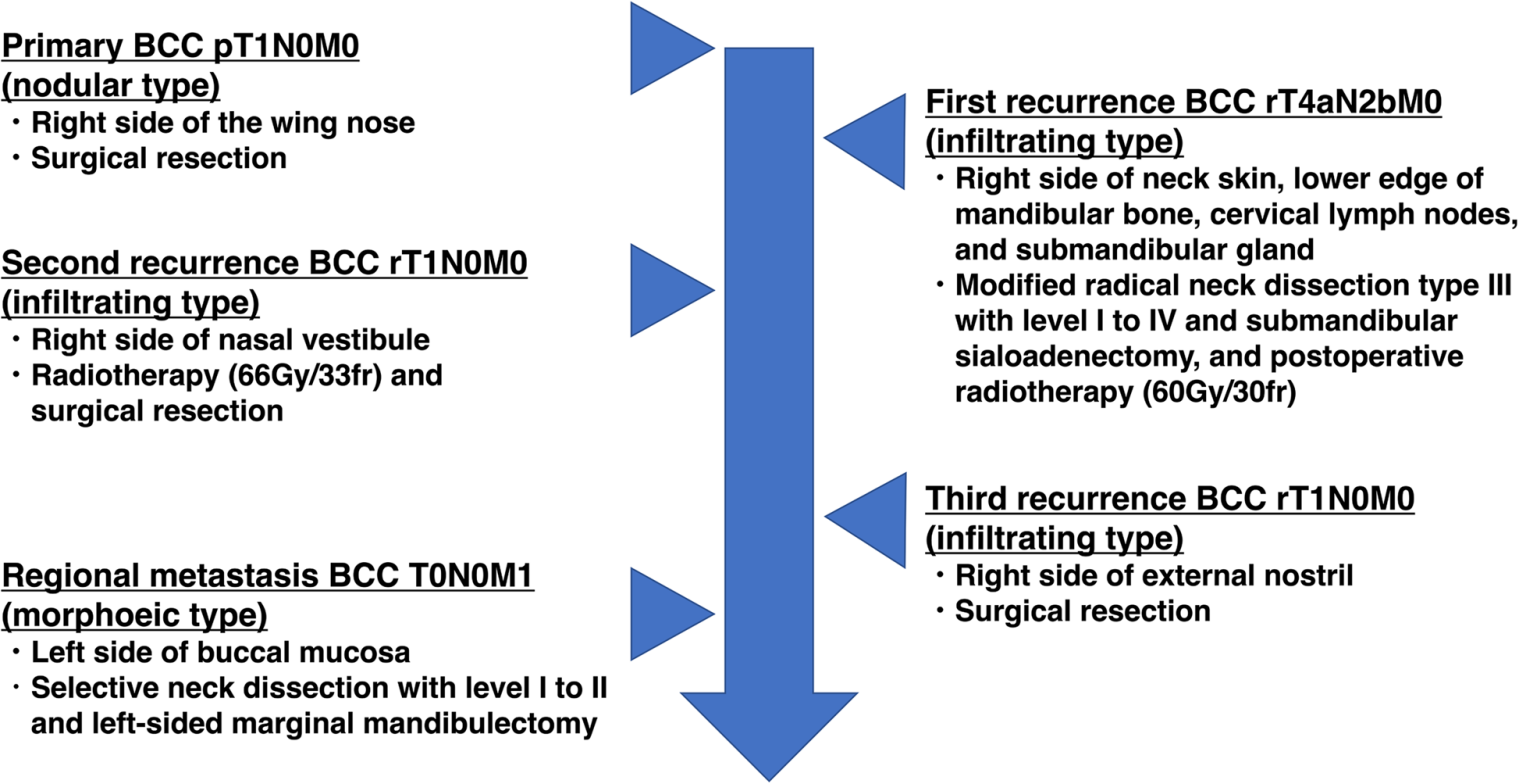
The course of this case. This patient developed primary basal cell carcinoma (BCC) in the right wing of the nose, and its histological finding was nodular subtype. After the treatment for primary BCC, he developed a total of three times local recurrence in the head and neck region, each of which was an infiltrating subtype. Finally, this patient developed metastasis in the buccal mucosal region which was on the opposite side of the primary and recurrent side and was the morphoeic subtype

**Fig. 2:**
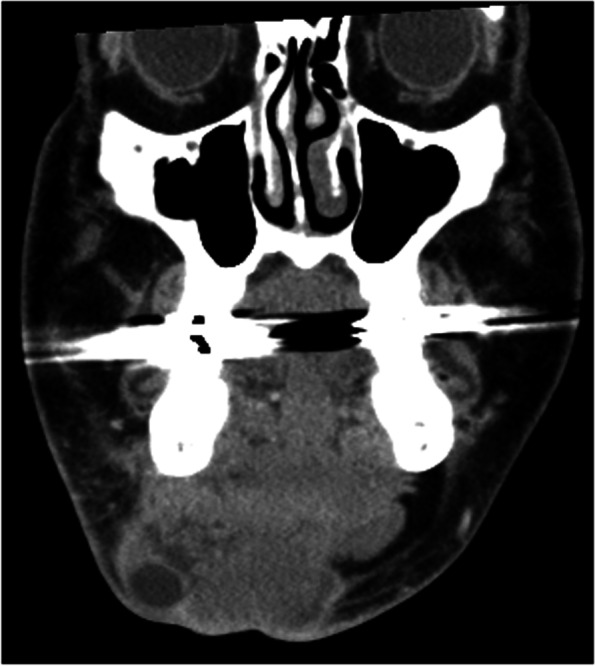
Coronal computed tomographic images of first recurrence indicated that the recurrent lesion was infiltrated into right submandibular glands and involving enlarged lymph nodes with peripheral rim enhancement

A 63-year-old Japanese man was referred to our department by an otolaryngologist because of swelling in the left lower gingiva. He had no symptoms except for trismus. His maximal interincisal distance was 30 mm. He ran a bicycle shop and had few opportunities for frequent exposure to ultraviolet rays and sunlight. He had smoked for 20 years (from the ages of 20–40 years) and had regularly drunk alcohol for 43 years. He had hypertension, hyperuricaemia and chronic kidney disease (eGFR=25) but no family history of skin cancer. The history of the present illness revealed that he had suffered multiple recurrent BCCs before presenting to our department (Fig. 1). A skin cancer on the right wing of the nose had been diagnosed and surgically resected. The lesion was resected in situ with a 2-mm negative margins. Histopathological examination revealed that the tumour was nodular BCC (pT1N0M0). He received regular observation once a month in the first year after the surgery and showed no signs or symptoms of recurrence or metastasis, and was extended the period to once a half-year from the second year after the surgery. Five years after the surgery, regular computed tomography (CT) unveiled that the cancer recurred in the skin of the right side of his neck, around the lower edge of the mandibular bone, on the right side of the cervical lymph nodes, and over the right submandibular gland (Fig. 2). He was diagnosed as rT4aN2bM0. Then, he underwent modified radical neck dissection type III with level I to IV, and right submandibular sialoadenectomy. Pathological examination revealed recurrent lesion with surgical margin positive and two positive nodes with ENE (total 2/26 including RIIa; 1/9 with ENE and RIII-1; 1/2); thus, he was carried out postoperative intensity modulated radiation therapy (IMRT) (total dose: 60 Gy in 30 fractions). Six years after the initial treatment, he suffered a second local recurrence on the right side of the nasal vestibule with the size of 9×10×9 mm. This time, he was diagnosed as rT1N0M0. Against 2nd recurrent lesion, he first was treated with radiotherapy (total dose: 66 Gy in 33 fractions) and finally underwent surgery. A third recurrence, occurring 7 years after the initial treatment, was on the side of the external right nostril, which was surgically resected. Its diagnosis was rT1N0M0 and the evaluation of surgical margin was negative. All the recurrent tumours exhibited infiltrating features in the pathological examinations.

**Fig. 3:**
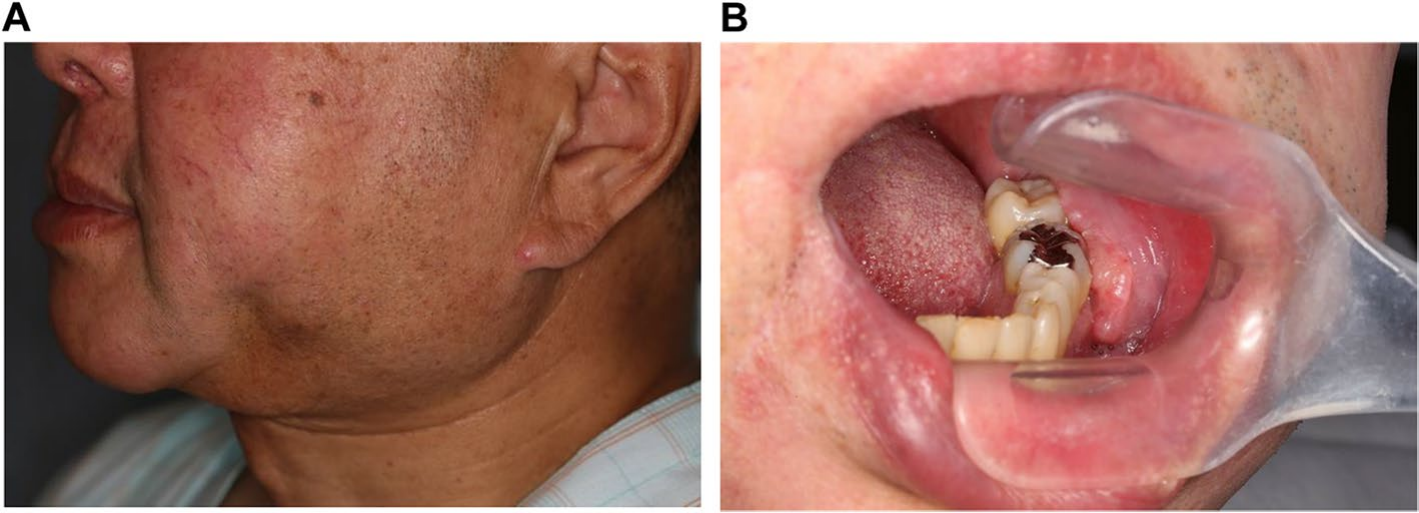
**A** Extraoral photograph shows a mass in the left buccal region. **B** The oral lesion was 33 × 12 mm in size with induration, and ulceration around the buccal mucosa and the lower gingiva was present

An extraoral examination revealed asymmetry of the face and swelling of the buccal region, this time on the left side, but no apparent enlarged cervical lymph nodes (Fig. 3A). An intraoral examination revealed that the lesion was 33 × 12 mm in size with induration that extended toward the buccal mucosa, and intractable ulceration was present around the buccal mucosa and the lower gingiva (Fig. 3B).

**Fig. 4:**
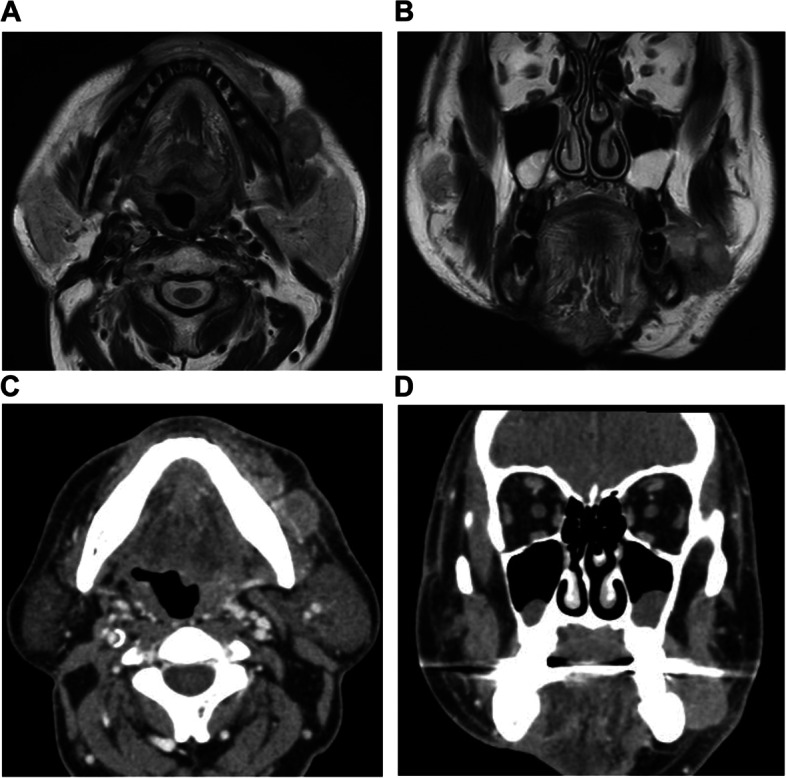
Axial (**A**) and coronal (**B**) T1-weighted magnetic resonance images and axial (**C**) and coronal (**D**) computed tomographic images, all of the head, depicted the lesion extending through the buccinator muscle toward the platysma muscle and involving the surface of the buccal skin

CT and magnetic resonance imaging depicted the lesion in the left buccal region, extending through the buccinator muscle toward the platysma muscle and reaching close to the surface of the buccal skin (Fig. 4A–D). Additionally, this lesion was also extended towards alveolar bones, and CT found the signs of osteolysis with the adjacent bone marrow exhibiting the significant signs of osteosclerosis. An incisional biopsy sample from the buccal lesion showed features of neoplasia: the formation of nests composed of basaloid-like epithelial cells underneath the oral epithelium and a palisading arrangement of the peripheral cells of the nests. Immunohistochemical (IHC) stains also revealed partial positivity for malignancy in the specimen. These pathological findings were indicative of metastatic BCC (pM1).

**Fig. 5:**
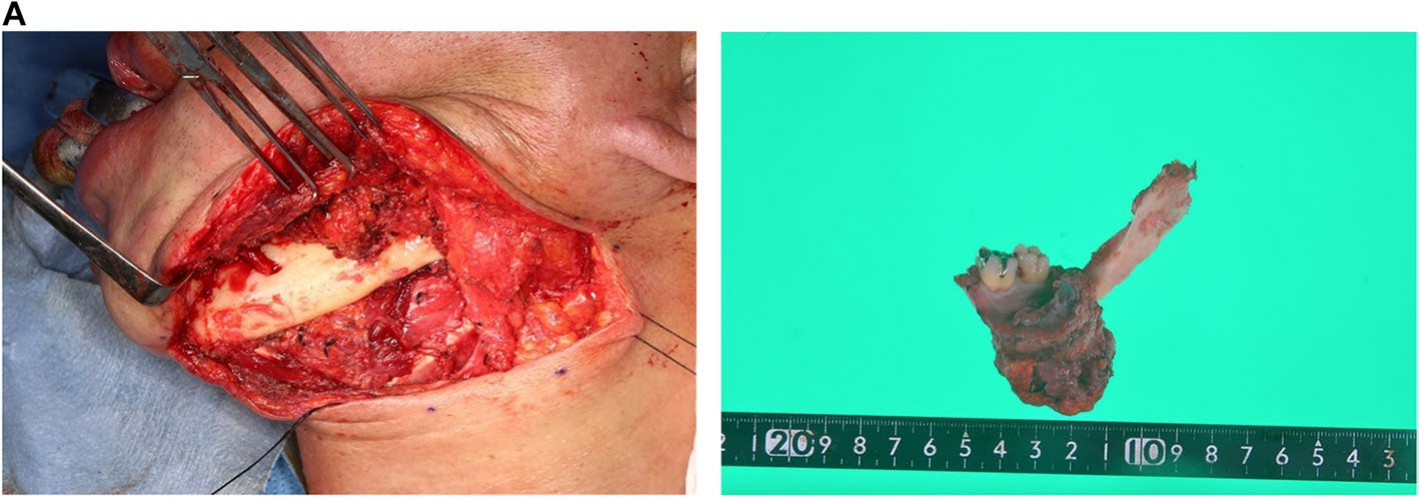
Metastatic lesion infiltrated the mandibular bone. Resection of the lesion involved the left side of the mandibular bone

**Fig. 6:**
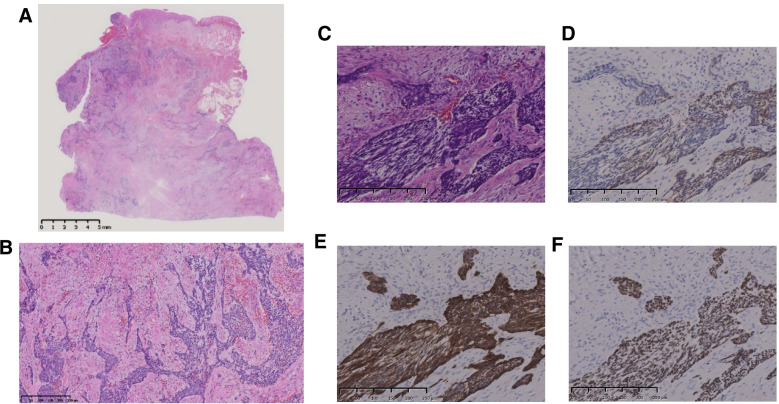
**A**–**C** Metastatic lesion exhibited several thin strands and nests of basaloid cells and was surrounded by dense stroma; these features were suggestive of the morphoeic subtype of basal cell carcinoma (BCC) (haematoxylin-eosin stains. **A** Original magnification 1×. **B** Original magnification 10×. **C** Original magnification 200×). **D**–**F** Immunohistochemical results. Ber-EP4 was expressed in the BCC cells (**D**, original magnification 200×). CK5/6 and p63 were expressed in the BCC cells (**E** and **F**, original magnification 200×, respectively)

**Fig. 7:**
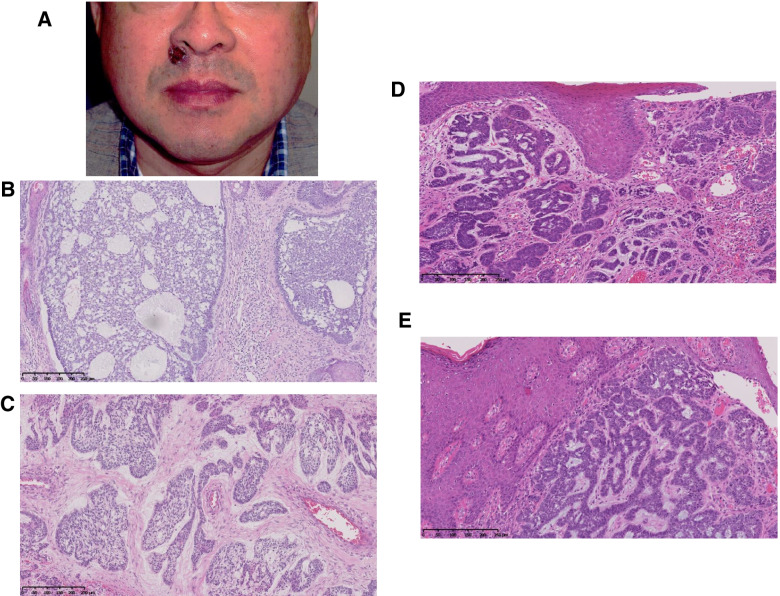
**A** Surface of the primary tumour was ulcerated around the right side of the nasal alae [primary basal cell carcinoma (BCC)]. **B** Pathological findings for the primary tumour indicated a nodular subtype of BCC. **C** Pathological findings for the first recurrent lesion developed in the right side of the neck and lower edge of the mandibular bone indicated an infiltrative subtype of BCC. **D** Pathological findings for the second recurrent lesion developed in the right side of the nasal vestibule indicated an infiltrative subtype of BCC. These findings were similar to those of the first recurrent lesion. **E** Pathological findings of the third recurrent lesion developed in the external right nostril indicated an infiltrative subtype of BCC. These findings were similar to those of the first and second recurrent lesions

One month after the biopsy, the patient underwent left-sided selective neck dissection with level I to II and left-sided marginal mandibulectomy. The lesion was resected in situ with a 5-mm safety margin, and 10 mm of the lower mandibular edge was left (Fig. 5). Pathological examination revealed that the tumour had infiltrated the musculature (Fig. 6A–C) with negative nodes. IHC stains were positive for cytokeratin (CK) 5/6, p63 and 34βE12; partially positive for CK AE1/AE3, Ber-EP4 and Bcl-2 protein; and negative for calponin, α-smooth muscle actin and epithelial membrane antigen (EMA) (Fig. 6D–F). The clinical history and pathological findings indicated a diagnosis of metastatic BCC (pT0N0M1) in the left buccal region that exhibited morphoeic features; this is a histologically uncommon type of primary BCC (Fig. 7A, B) and other recurrent BCCs (Fig. 7C–E).

Nine months after this surgery, he suffered a relapse in the same region. Because of his history of radiotherapy in the facial region, he additionally received palliative radiotherapy (total dose: 24 Gy in 12 fractions). In the 11 months since the radiotherapy was completed, the tumour size (18 × 8 mm) has remained stable. Given the patient’s treatment history and the tumour’s resistance to previous treatment, his prognosis is considered poor. He has refused additional treatment, and considering his history of radiotherapy and his renal dysfunction, it should not be reasonable to carry out surgery including reconstruction, and he is now being monitored regularly.

## Discussion

No metastatic BCC in the oral cavity has been reported previously. BCC commonly occurs as skin lesions, and intraoral BCC is extremely rare. BCC metastasizes via both lymphatic and hematogenous routes, and the average length of survival is documented as 3.6 years [4] and 8–14 months [5, 6], respectively. With metastases via the hematogenous route, the lungs (33%) and bone (20%) are the representative regions. Additionally, because intraoral BCC is extremely rare, and because the pathohistological findings of basaloid squamous cell carcinoma (basaloid SCC) and peripheral ameloblastoma are similar to those of BCC, establishing an accurate diagnosis is difficult.

Considering this case as primary or metastatic lesion, we diagnosed oral lesion as metastasis according to the criteria established by Lattes and Kessler [7]. These criteria are considered for the diagnosis of metastatic BCC including the following: (1) The primary tumour should arise in the skin and not in the mucosa. (2) Metastasis must be demonstrated at a site distant from the primary lesion and should not result from direct extension. (3) The histological picture should be the same in the primary and metastatic lesion. (4) The histology should confirm with that of BCC without a squamous cell component. In our case, metastasis was observed in the left side of the buccal mucosa and the lower gingiva, which occurred in distant region from primary and recurrent lesions. Additionally, this oral lesion did not show any squamous cell component. Regarding item 3, metastatic lesion had different histological subtype compared to primary lesion. As described in Fig. 1, the primary BCC in our patient was nodular, which is believed to be a relatively low-grade subtype of BCC. The relapse lesions on the side of the right external nostril and the right side of the chin were infiltrative, which is reportedly one of the most common histological subtypes associated with local relapse and metastasis [8]. The metastatic lesion in the left buccal mucosa exhibited morphoeic characteristics, which also represents a relatively high-grade subtype of BCC [8]. Given the patient’s clinical course and these pathological findings, we speculated that the phenotype of this tumour transformed, according to multiple relapses and metastasis. Therefore, these histological features and the criteria support our diagnosis of metastatic BCC.

Furthermore, as the histological features of BCC resembles some lesions including basaloid SCC, basal cell adenocarcinoma and other neoplasms, IHC staining can greatly help us lead to the exact diagnosis. Ber-EP4, a monoclonal antibody that identifies specific epithelial glycoprotein adhesion molecules (EpCAM), was found on BCC cells and has a high sensitivity and specificity in detecting only BCC cells. Thus, Ber-EP4 can rule out peripheral ameloblastoma which does not show any features of basal cells [9]. As for basaloid SCC, Ber-EP4 also stains positive in this neoplasm; however, histological criteria including focal squamous differentiation, a basaloid pattern associated with frank SCC or carcinoma in situ, can help us distinguish from BCC [10]. Additionally, basal cell adenocarcinoma, a rare salivary gland tumours with basaloid features, can distinguish from BCC by unique histological findings like a solid or cribriform pattern that mimics the feature of adenoid cystic carcinoma [11]. Besides the differences of histological findings, several markers like CK AE1/AE3, CK 5/6, p63 and EMA also help us distinguish the neoplasms that exhibit similar histological features from BCC. CK AE1/AE3 is a mixture of two different clones of anti-cytokeratin monoclonal antibodies, AE1 and AE3, both of which can detect certain high and low molecular weight keratins, respectively. This marker has been described as pancytokeratin and can be useful for confirming the specimen that originated from the epithelial tissue [12]. CK 5/6 are mainly expressed in keratinizing (epidermis) and nonkeratinizing (mucosa) squamous epithelium [13], and p63 is well known for the keratinocyte stem cell marker and consistently expressed in the basal cells and cutaneous appendages [14]. In this case, both of markers stained positive, which suggested that the oral lesion had a feature of cutaneous neoplasm. EMA normally expresses in glandular or luminal epithelial cells, which is usually employed to distinguish BCC (Ber-EP4+/EMA–) from SCC (Ber-EP4–/EMA+) [15]. In our patient, these IHCs suggested that the oral lesion originated from epithelial tissue and had a feature of cutaneous neoplasms. Additionally, the nest region of the specimen was Ber-EP4 and Bcl-2 positive and EMA negative specific in BCC. Considering these results, we concluded that the oral lesion was not primary intraoral BCC but the metastatic lesion of cutaneous BCC.

Once metastatic BCC in the oral cavity was described in 1992 [16] as a lesion in the mandibular alveolar ridge. In this case, the patient was first diagnosed as BCC of the right shoulder and got surgical resection. Six years after the initial treatment, this patient presented with oral lesion which was revealed as BCC with biopsy. However, the authors did not confirm Ber-EP4 positivity with IHC staining and thus, whether the disease was actually metastatic BCC is unclear. Thus, to establish the diagnosis of such a rare disease, the patient’s background and examination results must be examined thoroughly.

Treatment of primary BCC usually includes surgery and radiotherapy. Generally, surgery is the initial treatment for most cases, and radiotherapy is an alternative for unresectable tumours or patients who are medically unable to undergo surgery.

According to previous reports, definitive radiotherapy is an effective treatment for head and neck BCC [17], and radiotherapy is recommended in cases in which wide resection can cause cosmetic or functional issues [18].

However, these conventional methods have rarely cured metastatic BCC. Two targeted therapeutic agents, vismodegib and sonidegib, which inhibit the hedgehog pathway, were recently approved by the US Food and Drug Administration for the treatment of locally advanced and metastatic BCC. Although these drugs have shown some benefit (objective response rate was 47.6% for vismodegib and 60.6% for sonidegib) [19], they have not improved the prognosis dramatically, and further studies would be required. In this case, since these agents have yet to be approved in Japan and considering his renal dysfunction, our patient could not have received them. The prognosis of metastatic BCC is reportedly poor, and the length of survival depends on where the metastatic lesion develops. The median length of survival is 8 months in patients with distant metastases but 3.5 years in those with metastasis in the lymph nodes [3, 20]. In our patient, metastasis was limited to the facial region, which tends to have a better prognosis than does distant metastasis. However, he had suffered from several local relapses and metastasis, which could have resulted from certain factors associated with high-grade phenotypes [21], including aggressive subtype, a history of radiotherapy and a history of local recurrence. Because of the rarity of metastasis, it is hard to anticipate the prognosis of this case, which is the limitation of this report.

In managing BCC, the tumour subtype and risk factors of high-grade malignancy should be considered. Our patient suffered a relapse in the left buccal mucosa and underwent palliative radiotherapy, and now, 11 months after radiotherapy, he is in stable condition and being monitored regularly.

## Conclusions

We reported a rare case of BCC in which a series of local relapses culminated in metastasis into the oral cavity 10 years after the first diagnosis of cutaneous BCC. We performed surgical resection and palliative radiotherapy, and the lesion is still being observed. Through this rare case, we realize that BCC that recurs and metastasizes tends to be difficult to control; thus, managing this cancer requires an understanding of the tumour properties according to histopathological findings and consideration of the patient’s histories of radiotherapy and relapse.

## Data Availability

Not applicable.

## Abbreviations

BCC: Basal cell carcinoma
IHC: Immunohistochemical
